# Intestinal Lipoma Acting as a Lead Point of Intussusception: A Case Series

**DOI:** 10.2174/0115734056337435250206100026

**Published:** 2025-02-18

**Authors:** Mei-Ying Jiang, Xiao-Yan Luo, Xiu-Qin Luo, Ai-fang Jin, Zhe-Huang Luo

**Affiliations:** 1 Department of Nuclear Medicine, Jiangxi Provincial People’s Hospital, The First Affiliated Hospital of Nanchang Medical College, Nanchang, China; 2 Department of Clinical Laboratory, Jiangxi Provincial Children’s Hospital. The Affiliated Children’s Hospital of Nanchang Medical College, Nanchang, China; 3 Cardio-Thoracic Surgery, Jiangxi Provincial People’s Hospital, The First Affiliated Hospital of Nanchang Medical College, Nanchang, China

**Keywords:** Lipoma, Intussusception, Fluorodeoxyglucose, Positron emission tomography/computed tomography, Benign tumor, Malignant tumor

## Abstract

**Background:**

Lipomas represent a rare benign etiology of intussusception in adults, affecting both the small intestine and the colon. Diagnosing intussusception in adults can be challenging, and there are no reports on the use of positron emission tomography/CT (PET/CT) in the diagnosis of lipoma-induced intussusception. This study aimed to preliminarily explore the potential diagnostic utility of ^18^F-FDG PET/CT in the diagnosis of intussusception caused by lipomas.

**Methods:**

We conducted a retrospective review of the clinical characteristics and imaging findings of three patients diagnosed with lipoma-induced intussusception using ^18^F-FDG PET/CT from 2019 to 2023 at our hospital.

**Results:**

The three cases presented with diverse clinical presentations and were diagnosed based on PET/CT imaging. Surgical confirmation was obtained in two cases. Lipomas were identified in both the small intestine and the colon, with one case displaying increased metabolic activity on FDG uptake, suggesting a possible link between FDG uptake and clinical severity.

**Conclusion:**

Lipoma is a benign cause of intussusception that can occur in both the small intestine and the colon. The symptoms of adult intussusception are often atypical and variable. Imaging modalities, particularly PET/CT, are instrumental in diagnosing intussusception due to lipomas, differentiating between benign and malignant causes, and assessing the severity to inform treatment strategies.

## 1 INTRODUCTION

Intussusception is a condition wherein a proximal segment of the bowel telescopes into the lumen of an adjacent distal segment, thereby obstructing the normal flow of intestinal contents. While it is a common occurrence in children, intussusception in adults is relatively infrequent and is often triggered by an underlying mass, frequently malignant, which acts as a lead point [1]. Neoplastic intussusception is commonly detected in the right half of the colon and typically involves only a single segment [2]. Gastrointestinal lipomas, especially giant ones (>4 cm), are the most prevalent benign tumors causing intussusception in adults [3]. The presentation of intussusception in adults can vary from being asymptomatic to exhibiting symptoms of complete bowel obstruction, necessitating urgent surgical intervention. Diagnosing intussusception in adults is challenging because the classic clinical triad seen in children, such as abdominal pain, a palpable abdominal mass, and blood in the stool, is often absent. Computed tomography (CT) imaging plays a crucial role in identifying the underlying etiology [4, 5].

To date, no studies have investigated the use of positron emission tomography/computed tomography (PET/CT) in the diagnosis of intussusception caused by lipomas. This report describes three cases of intussusception due to lipomas that were diagnosed using 18F-FDG PET/CT imaging.

## 2 METHODS AND MATERIALS

The Materials and Methods section explicitly states the source of the case data and patients' informed consent.

In this retrospective study, we evaluated the clinical characteristics and imaging manifestations of three patients diagnosed with lipoma-induced intussusception using CT or 18F-FDG PET/CT at the First Affiliated Hospital of Nanchang Medical College from 2019 to 2023. The diagnosis of intussusception caused by lipoma was established in two patients based on PET/CT imaging and subsequently confirmed through surgical intervention. In the remaining patient, the diagnosis was inferred from characteristic CT imaging features, including the sleeve sheath sign and the concentric circle sign.

All patients provided informed consent for the anonymized use of their clinical data and imaging for publication purposes. Given the retrospective nature of this study, the institutional review board waived the requirement for approval.

## 3 RESULT

### 3.1 Case 1

**Fig. (1):**
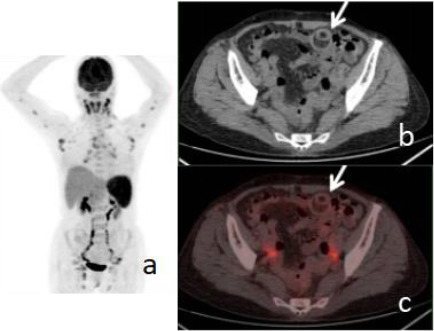
18F-FDG PET/CT of Case 1 showing intussusception due to an ileal lipoma. Multiple lymphadenopathy and splenomegaly with FDG avidity were shown in PET/MIP (**a**). Double sign (arrows) shown in CT (**b**) and fusion image (**c**).

A 67-year-old female presented to our institution with a persistent fever of unknown origin for a PET/CT scan. Approximately two weeks prior, she had experienced a fever that reached a peak of 40.0°C, associated with chills and an aversion to cold, but without accompanying headache, dizziness, nausea, or vomiting. A chest CT scan performed at a local hospital yielded unremarkable findings. An unenhanced abdominal CT scan identified multiple lipomas within the small bowel. Despite treatment with broad-spectrum antibiotics, her fever persisted, leading to a referral to our hospital for further investigation. Upon admission, her body temperature was 39°C, while her blood pressure, heart rate, and respiratory rate were within normal ranges. Laboratory findings included: C-reactive protein 50.20 mg/L (normal: 0-10 mg/L), ferritin 949.00 ng/ml (normal: 13-150 ng/ml), alpha-fetoprotein 0.9 ng/ml (normal: 0-7 ng/ml), carcinoembryonic antigen 2.46 ng/mL (normal: 0-6.5 ng/mL), and carbohydrate antigen 19-9 6.50 U/mL (normal: 0-27 U/mL). The subsequent PET/CT scan revealed multiple lymphadenopathies and splenomegaly with increased FDG uptake, in addition to the presence of multiple small intestinal lipomas. The scan also demonstrated a characteristic concentric circle sign within the abdominal intestinal lumen, indicative of intussusception (Fig. **1**). The affected intestinal segment exhibited no significant FDG uptake, and no suspicious foci of increased metabolic activity were observed in the distal segment, effectively ruling out malignant intestinal tumors. Surgical intervention confirmed a lipoma as the leading point of the intussusception, measuring approximately 17 mm in diameter. There was no evidence of significant hyperemia or edema in the intussuscepted intestinal segment. A biopsy of a right inguinal lymph node revealed reactive lymphoid hyperplasia.

### 3.2 Case 2

**Fig. (2):**
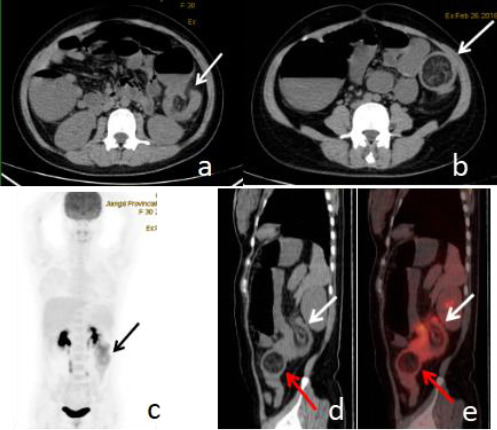
18F-FDG PET/CT of Case 2 showing intussusception due to colonic lipoma. Axial CT showing intussusception (**a**, arrow) and lipoma (**b**, arrow). PET MIP image (**c**) showing hypermetabolic lesion in the left mid-abdomen (arrow). Saggital CT (**d**) and fusion image (**e**) showing intussusception (white arrows) and lipoma (red arrows).

A 30-year-old female was admitted to our hospital with a 20-day history of episodic pain in the left lower quadrant and abdominal distension. Her abdominal discomfort had escalated, and she had experienced neither flatulence nor bowel movement for the past three days. Physical examination showed that the abdomen was flat and soft without tenderness and rebound pain. Laboratory investigations, including complete blood count, CA 19-9, and carcinoembryonic antigen (CEA) levels, were all within normal ranges. An abdominal CT scan identified a lipoma in the descending colon. To rule out other potential pathologies, a preoperative whole-body PET-CT scan was conducted, which revealed an intussusception with the lipoma serving as the leading point (Fig. **2**). The involved intestinal segment demonstrated increased FDG uptake. Three days thereafter, the patient underwent surgical intervention, which confirmed the preoperative imaging diagnosis. Histopathological analysis of the resected mass confirmed a submucosal lipoma with a diameter of 4 cm. Notably, the intussuscepted intestinal segment exhibited marked hyperemia and edema.

### 3.3 Case 3

**Fig. (3):**
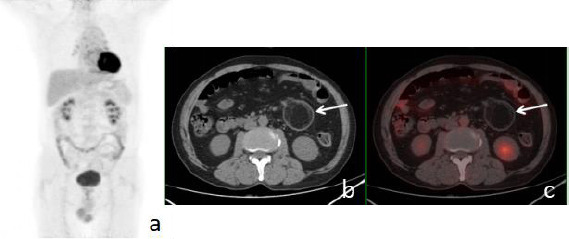
18F-FDG PET/CT of Case 3 showing jejunal lipoma. Abdominal CT before 5 days revealed a small intestinal intussusception, but the PET/CT showed it was revolved. No malignancy was obeserved. (**a**), PET MIP image; (**b**), Axial CT; (**c**), axial PET/CT fusion image.

A 69-year-old male with a facial mass was referred to our institution for a PET/CT assessment. The mass was initially suspected to be a metastatic lesion at a local hospital. Standard laboratory tests, including complete blood counts, liver and kidney function panels, and serum tumor marker levels, were all within normal reference ranges. An abdominal CT scan identified a lipoma as the leading point of an intussusception, featuring the classic sleeve sheath and concentric circle signs. The PET/CT scan revealed a hypometabolic mass in the left temporal fossa, which was subsequently surgically excised and confirmed to be a meningioma. Additionally, a lipoma without FDG uptake was detected in the small intestine (Fig. **3**). The corresponding intestinal segment did not exhibit increased FDG uptake.

### 3.4 Findings Summary

This retrospective analysis encompasses three patients (one male and two females) with an age range of 30 to 69 years. Among these, two patients harbored solitary intestinal lipomas, whereas the remaining patient had multiple lipomas, with the small intestine being involved in two instances and the large bowel in one. Intussusception was diagnosed in one patient due to abdominal pain, whereas it was incidentally detected during unrelated examinations in the other two patients. The symptomatic patient’s intussusception exhibited increased metabolic activity, as evidenced by FDG uptake. In contrast, the two asymptomatic patients’ intussusceptions did not demonstrate significant FDG uptake, implying a potential link between FDG uptake and the clinical severity of intussusception. No FDG uptake was detected in any of the lipomas. The clinical and laboratory features of these patients are summarized in Table **1**.

### 3.5 Follow up

All patients were followed up for a duration ranging from 7 to 13 months. Patient 1 remained under surveillance and reported occasional episodes of abdominal pain; however, there was no recurrence of intussusception or development of intestinal obstruction. Patient 2 had an uneventful recovery postoperatively and did not encounter any complications during the follow-up period. Patient 3, who was managed conservatively without surgical intervention, remained free of complications related to intestinal obstruction or intussusception throughout the follow-up duration.

## 4 DISCUSSION

Intussusception presents in various forms, including jejuno-jejunal, jejuno-ileal, ileo-colonic, and, less frequently, colo-colic types. In adults, intussusception caused by lipomas is a rare occurrence [6, 7]. This report delineates three cases where lipomas served as the lead points for intussusception, identified through CT or ^18^F-FDG PET/CT imaging. To the best of our knowledge, the detection of intussusception with lipomas as the leading point using ^18^F-FDG PET/CT has not been previously reported. The management and follow-up of these cases suggest that PET/CT imaging may be a valuable tool for assessing the severity of intussusception and guiding the selection of treatment modalities.

Intussusception typically occurs in the more mobile and redundant segments of the intestine, such as the jejunum, ileum, and colon, and is less commonly observed in the anatomically fixed upper digestive tract, including the esophagus, stomach, and duodenum. Although more prevalent in children, intussusception is less frequent in adults, with the small intestine being the most common site of intussusception in adults, often associated with a lead point [8]. The majority of adult small intestinal pathologic lead points are benign, although some, such as diffuse metastases, can be malignant. Conversely, when a lead point is present in adult colonic intussusception, it is most often due to a malignant tumor [9]. Gastrointestinal lipomas are typically asymptomatic and are often discovered incidentally. The incidence of gastrointestinal lipomas has been reported to range from 0.15% to 4.4% [10, 11]. These lipomas are more commonly found in the colon of patients over 50 years of age [12]; two of the cases in our series were over this age threshold. Approximately 90% of adult intussusceptions have pathogenesis involving a lead point [13], with lipomas accounting for 5% of all adult intussusception cases [9].

Colonic lipomas are the most common benign etiology of colonic intussusception. As the second most frequent benign tumor in the colon, following adenomas, lipomas are most commonly detected in the cecum and ascending colon. Colonic lipomas vary in size, with those larger than 4 cm generally classified as giant lipomas [14]. Small colonic lipomas are usually asymptomatic, whereas large colonic lipomas may present with symptoms, such as abdominal pain, bloating, changes in bowel habits, and, rarely, bleeding and intussusception, as observed in Case 2. It has been reported that most symptomatic colonic lipomas exceed 2 cm in diameter, and the lipoma in Case 2 was 4 cm in diameter.

Small intestinal lipomas constitute approximately 25% of gastrointestinal lipomas and are found in the submucosa in 90% of patients. Unlike the female predilection for colonic lipomas, there is a male predominance in patients with symptomatic small intestinal lipomas [15]. Benign lesions are more commonly the lead point in small intestinal intussusceptions. Small intestinal lipomas are less common and less likely to cause intussusceptions compared to colonic lipomas. These lipomas can be solitary or multiple [16], and the narrower lumen of the small intestine allows even a smaller lipoma to precipitate intussusception, as seen in Case 1.

Adult intussusception presents with a broad spectrum of clinical manifestations, which can complicate diagnosis, unlike in children. Among our three patients, only one reported abdominal pain. Imaging, such as abdominal CT, is crucial for diagnosing adult intussusception [17]. There is a paucity of studies on the application of 18F-FDG PET/CT for intussusception. Intussusception may be incidentally detected on PET/CT in patients with neoplasms [18], as in Cases 1 and 3. The advantages of PET/CT in adult intussusception are evident in its ability to delineate the apex, neck, intussusceptum, and intussuscipiens, as well as to determine whether the lead point lesion is benign or malignant. Gastrointestinal lipomas have characteristic CT features and do not exhibit FDG-avidity, preventing misdiagnosis. Inflamed intestinal segments can exhibit FDG-avidity in the intestinal wall, allowing PET/CT imaging to also assess the severity of intussusception and guide the selection of treatment approaches. A few intussusception tests have been completed with PET/CT. However, further experience needs to be gained.

## CONCLUSION

Lipomas represent a benign etiology of intussusception, which can occur within both the small intestine and the colon. The clinical presentation of adult intussusception is often atypical and diverse. Imaging modalities are pivotal in the diagnosis of lipoma-induced intussusception. Currently, PET/CT is infrequently employed in the assessment of this condition. PET/CT imaging not only delineates the presence of intussusception but also differentiates between benign and malignant lead point lesions. Additionally, the ability of inflamed intestinal segments to exhibit FDG-avidity allows PET/CT to evaluate the severity of intussusception due to lipomas and to inform the choice of treatment strategies.

## Figures and Tables

**Table 1:** Clinical features and image findings of the three patients with intussusception caused by a lipoma.

**CASE**	**1**	**2**	**3**
Sex	F	F	M
Age (years)	67	30	69
Abdominal pain	no	yes	no
Palpable mass	no	no	no
Fecal occult blood	no	no	no
CRP (mg/L)	50.2	7.4	4.8
CEA (ng/ml)	0-6.5	0-6.5	0-6.5
CA199 (U/mL)	0-27	0-27	0-27
Location of lipoma	Jejunoileum	Colon	Jejunum
Number of lipomas	Multiple	Single	Single
Size of lipoma (mm)	12-27	42	37
Treatment of intussusception	Operation	Operation	Spontaneously resolved
Outcome	No recurrence	No recurrence	No recurrence

**Note**: F, female; M, male.

## Data Availability

The data and supportive information are available within the article.

## LIST OF ABBREVIATIONS

CT: Computed Tomograpy
PET/CT: Positron Emission Tomography/computed Tomograpy
CEA: Carcinoembryonic Antigen

## STANDARDS OF REPORTING

STROBE guidelines were followed.
